# Systematic Review on the Effectiveness and Outcomes of Nivolumab Treatment Schemes in Advanced and Metastatic Cervical Cancer

**DOI:** 10.3390/diseases12040077

**Published:** 2024-04-15

**Authors:** Ion Petre, Corina Vernic, Izabella Petre, Cristian Sebastian Vlad, Simona Ioana Sipos, Anca Bordianu, Marc Luciana, Radu Dumitru Dragomir, Camelia Melania Fizedean, Cristina Vlad Daliborca

**Affiliations:** 1 Doctoral School, “Victor Babes” University of Medicine and Pharmacy Timisoara, Eftimie Murgu Square 2, 300041 Timisoara, Romania; petre.ion@umft.ro; 2 Department of Functional Sciences, Medical Informatics and Biostatistics Discipline, “Victor Babes” University of Medicine and Pharmacy Timisoara, Eftimie Murgu Square 2, 300041 Timisoara, Romania; cvernic@umft.ro; 3 Department XII of Obstetrics and Gynecology, “Victor Babes” University of Medicine and Pharmacy Timisoara, Eftimie Murgu Square 2, 300041 Timisoara, Romania; petre.izabella@umft.ro; 4 Department of Biochemistry and Pharmacology, “Victor Babes” University of Medicine and Pharmacy Timisoara, Eftimie Murgu Square 2, 300041 Timisoara, Romania; vlad.cristian@umft.ro (C.S.V.); sipos.simona@umft.ro (S.I.S.); vlad.daliborca@umft.ro (C.V.D.); 5 Department of Plastic Surgery and Reconstructive Microsurgery Bagdasar-Arseni, Emergency Hospital Bucharest, University of Medicine and Pharmacy “Carol Davila”, 010825 Bucharest, Romania; 6 Department VII of Internal Medicine II, Division of Nephrology, “Victor Babes” University of Medicine and Pharmacy Timisoara, Eftimie Murgu Square 2, 300041 Timisoara, Romania; marc.luciana@umft.ro; 7 Department of Oncology, “Victor Babes” University of Medicine and Pharmacy Timisoara, Eftimie Murgu Square 2, 300041 Timisoara, Romania; dragomirradu91@gmail.com; 8 Methodological and Infectious Diseases Research Center, “Victor Babes” University of Medicine and Pharmacy Timisoara, Eftimie Murgu Square 2, 300041 Timisoara, Romania

**Keywords:** gynecology, oncology, cervical cancer

## Abstract

Advanced and metastatic cervical cancer remains a formidable challenge in oncology, with immune checkpoint inhibitors such as the PD-1 inhibitor nivolumab emerging as a potential therapeutic option. This systematic review rigorously assesses the effectiveness and outcomes of various nivolumab treatment regimens within this patient cohort, drawing from clinical trials and real-world evidence up to December 2023. Following a comprehensive search across PubMed, Scopus, and Embase, four studies were deemed eligible, involving a collective total of 80 patients. One preliminary trial data were excluded from the final analysis, as well as four other proceedings and abstracts on the efficacy and safety of nivolumab on advanced cervical cancer. The patients’ average age across these studies was 48 years, with an average of 38% having an Eastern Cooperative Oncology Group (ECOG) performance status of 1. Notably, 64% of all patients were positive for high-risk HPV, and 71% exhibited PD-L1 positivity, indicating a substantial target population for nivolumab. The analysis revealed a pooled objective response rate (ORR) of 48%, with a disease control rate (DCR) averaging 71%. Moreover, progression-free survival (PFS) at 6 months was observed at an average rate of 50%, reflecting the significant potential of nivolumab in managing advanced stages of the disease. The review highlights the influence of PD-L1 status on response rates and underscores the enhanced outcomes associated with combination therapy approaches. By delineating the variability in treatment efficacy and pinpointing key factors affecting therapeutic response and survival, this systematic review calls for further investigations to refine nivolumab’s clinical application, aiming to improve patient outcomes in advanced and metastatic cervical cancer.

## 1. Introduction

Cervical cancer remains a significant public health concern worldwide, with epidemiological data indicating it as the fourth most common cancer among women globally [1,2]. Despite advancements in screening and vaccination efforts aimed at reducing the incidence of human papillomavirus (HPV), the primary causative agent, cervical cancer continues to present substantial morbidity and mortality rates, particularly in low- and middle-income countries [3,4,5]. In 2020, the World Health Organization reported approximately 600,000 new cases of cervical cancer and more than 300,000 deaths, underscoring the ongoing challenge it poses to global health systems [6,7].

The treatment landscape for advanced cervical cancer has evolved significantly over the past decade, with the introduction of targeted therapies and immune checkpoint inhibitors marking a paradigm shift in management approaches [8,9,10]. Nivolumab, a programmed death-1 (PD-1) inhibitor, has emerged as a promising option, offering a new therapeutic avenue for patients with advanced disease [11]. By modulating the immune response, nivolumab has shown potential in improving outcomes for a subset of patients, yet its role and efficacy within the broader context of cervical cancer treatment remain to be fully elucidated [12].

Recent clinical trials and real-world studies have begun to shed light on the effectiveness and safety profiles of immune checkpoint inhibitors (ICIs) in the treatment of advanced cancer, including metastatic cancer [13]. However, the heterogeneity in treatment regimens, patient populations, and study outcomes presents challenges in interpreting and generalizing these findings [14]. Moreover, as the healthcare community continues to prioritize personalized medicine, understanding the variability in patient responses to PD-1 treatments becomes increasingly important [15]. Factors such as biomarker expression, tumor microenvironment, and previous treatment histories may influence treatment outcomes depending on the PD-1 molecule, necessitating a detailed review of the evidence to guide clinical decision-making and optimize patient care [16].

Therefore, this study aims to systematically review the literature on nivolumab treatment schemes for advanced and metastatic cervical cancer, focusing on the effectiveness and outcomes of different therapeutic regimens. By evaluating clinical trial data, real-world evidence, and meta-analyses, the study seeks to provide a comprehensive overview of nivolumab’s role in treating advanced cervical cancer, identify potential predictors of response, and highlight areas requiring further investigation.

## 2. Materials and Methods

### 2.1. Protocol and Registration

To conduct a systematic review of the literature on the effectiveness and outcomes of nivolumab treatment schemes in patients with advanced cervical cancer, this study implemented an exhaustive search strategy across major electronic databases, including PubMed, Scopus, and Embase. The literature search aimed to include publications up to December 2023, ensuring that the review captures the most up-to-date and relevant studies on the subject.

The search strategy employs a wide range of keywords and phrases directly related to the study’s objectives, with a focus on the specific therapeutic role of nivolumab. Key search terms include the following: “advanced cervical cancer”, “cervical cancer”, “metastatic cancer”, “cervix”, “nivolumab”, “PD-1 inhibitor”, “immune checkpoint inhibitor”, “treatment outcomes”, “clinical effectiveness”, “combination therapy”, “monotherapy”, “adverse events”, “survival rates”, “quality of life”, “treatment regimens”, “response rates”, “progression-free survival”, “overall survival”, and “treatment-related toxicity”.

To ensure thorough and efficient literature retrieval, Boolean operators (AND, OR, NOT) were used to effectively combine and refine search terms. The search string was constructed as follows: ((“advanced cervical cancer” OR “cervical cancer” OR “metastatic cervical cancer” OR “cancer of the cervix”) AND (“nivolumab” OR “PD-1 inhibitor” OR “immune checkpoint inhibitor”) AND (“treatment outcomes” OR “clinical effectiveness” OR “combination therapy” OR “monotherapy”) AND (“survival rates” OR “quality of life” OR “response rates” OR “progression-free survival” OR “overall survival”) AND (“adverse events” OR “treatment-related toxicity”)).

Adhering to the Preferred Reporting Items for Systematic Reviews and Meta-Analyses (PRISMA) guidelines [17], this protocol was crafted to ensure a structured, transparent, and reproducible methodology. To promote the accessibility and transparency of our research process and findings, this review has been registered with the Open Science Framework, providing open access to our methodologies and anticipated outcomes. The registration code for this review is osf.io/q23cb.

### 2.2. Inclusion and Exclusion Criteria

For the systematic review on the effectiveness and outcomes of nivolumab treatment schemes in advanced and metastatic cervical cancer, studies were selected based on a set of meticulously defined inclusion and exclusion criteria to ensure the analysis was both comprehensive and relevant. To be included, studies had to involve patients diagnosed with advanced (stages IB2-IVA according to FIGO staging or equivalent) or metastatic cervical cancer, treated with nivolumab either as monotherapy or in combination with other therapeutic agents. These studies spanned all age groups and genders and explicitly examined the outcomes of nivolumab treatment, focusing on clinical effectiveness, survival outcomes such as overall survival and progression-free survival, response rates, quality of life, and treatment-related adverse events. A variety of study designs were considered, including randomized controlled trials, observational studies, clinical trials, cohort studies, case-control studies, cross-sectional studies, systematic reviews, and meta-analyses, provided they used validated instruments or clearly defined parameters to assess the specified outcomes.

Conversely, the review excluded studies not involving human participants, such as in vitro or animal model studies, to concentrate exclusively on human patient experiences and outcomes. Additionally, studies that did not specifically examine patients with advanced or metastatic cervical cancer undergoing nivolumab treatment or failed to differentiate the impact of nivolumab from other treatments in this specific patient population were omitted. The exclusion criteria also extended to studies that lacked clear, quantifiable outcomes related to the treatment effectiveness, survival rates, response rates, quality of life, or treatment-related adverse events, as well as those lacking sufficient detail for a comprehensive analysis. Furthermore, to maintain the credibility and reliability of the data included in the review, gray literature including non-peer-reviewed articles, preprints, conference proceedings, general reviews, commentaries, and editorials were excluded. Only peer-reviewed articles published in English were incorporated to ensure the thoroughness of the review and analysis. Through these stringent criteria, the review aimed to encapsulate high-quality, relevant evidence concerning the clinical utility, patient outcomes, and safety profile of nivolumab in the treatment of advanced and metastatic cervical cancer.

### 2.3. Definitions

In this systematic review, the terms “advanced” and “metastatic” cervical cancer are precisely defined to capture the specific patient populations under investigation, in accordance with established guidelines. Advanced cervical cancer refers to stages IB2 to IVA of the disease, as classified by the International Federation of Gynecology and Obstetrics (FIGO) [18]. This categorization encompasses cancer that has extended beyond the cervix but has not spread to distant organs. Specifically, it includes tumors larger than 4 cm in diameter (stage IB2) and those extending to adjacent regions such as the vagina or parametria (stages II and III), up to cancer that has invaded the bladder or rectum without spreading to distant sites (stage IVA). Metastatic cervical cancer, on the other hand, corresponds to stage IVB according to the FIGO staging system.

Programmed death-1 (PD-1) is a protein found on the surface of T cells, which plays a critical role in downregulating the immune system by preventing the activation of T-cells, thus contributing to self-tolerance and protection against autoimmunity [19]. PD-1 acts by binding to its ligands, PD-L1 and PD-L2, which are expressed on the surface of some tumor cells and other cells within the tumor microenvironment.

Immune checkpoint inhibitors (ICIs) are a class of drugs designed to block checkpoint proteins from binding with their partner proteins, thereby preventing the “off” signal from being sent to the immune system. By inhibiting these checkpoints, such as PD-1, ICIs enhance the immune system’s ability to detect and destroy cancer cells. Nivolumab is an example of a PD-1 inhibitor, a type of ICI, which works by blocking the interaction between PD-1 and its ligands, thereby promoting an immune response against cancer cells. ICIs have emerged as a significant advancement in cancer therapy, offering new treatment options for various types of cancer, including advanced and metastatic cervical cancer.

### 2.4. Data Collection Protocol

Initially, the search across designated electronic databases resulted in the identification of 702 articles. Following the initial retrieval, 149 duplicate entries were removed, ensuring each study was unique for the preliminary screening phase. Subsequently, two independent reviewers conducted a screening of the abstracts and titles based on the predefined inclusion and exclusion criteria detailed in the study protocol, focusing on studies involving nivolumab treatment in the specified patient population. The discrepancies between the reviewers at this stage were resolved through a structured discussion, and if consensus could not be reached, a third reviewer was consulted to make a final determination.

After the abstract screening, 211 articles were excluded for not meeting the inclusion criteria, primarily due to focusing on unrelated treatment methods, involving non-human subjects, or not specifying outcomes relevant to the effectiveness and outcomes of nivolumab treatment. The remaining articles underwent a full-text review for a detailed assessment against the study’s eligibility criteria. This comprehensive review further narrowed the selection to 62 articles deemed potentially suitable for inclusion in the final analysis.

**Figure 1:**
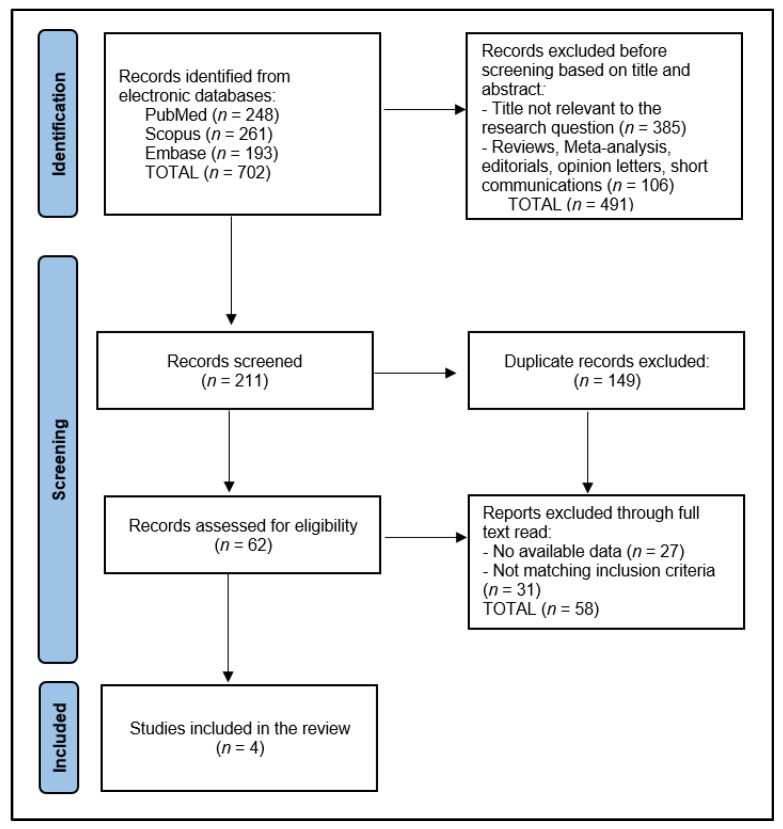
PRISMA Flow Diagram.

The final step involved a detailed evaluation of these articles by scrutinizing the study design, population characteristics, treatment regimens, and reported outcomes in line with the systematic review’s objectives. After careful consideration, 58 articles were excluded for various reasons, including insufficient data on treatment outcomes, a lack of clarity regarding the study design or methodology, and the duplication of data in multiple publications. Ultimately, 4 relevant studies were identified for inclusion in the systematic review, as presented in Figure 1.

### 2.5. Quality Assessment

For assessing study quality and bias risk, our review applied a dual method, blending qualitative and quantitative analyses. Observational studies’ quality was gauged using the Newcastle–Ottawa Scale [20], focusing on group selection, group comparability, and outcome or exposure assessment. The studies received a cumulative star score, categorizing their quality as low, medium, or high, enabling precise quality assessments. Two independent researchers evaluated each study, with any disagreements resolved via discussion or a third reviewer’s input.

## 3. Results

### 3.1. Study Characteristics

The final analysis examined the characteristics of four studies [21,22,23,24], as outlined in Table 1, conducted within the timeframe of 2019 to 2023. Each of these studies employed a clinical trial design, with two studies (Naumann et al. [22] and Santin et al. [23]) based in the United States. All studies reviewed, including Tamura et al. [21] from Japan, Naumann et al. [22], Santin et al. [23] from the United States, and Rodrigues et al. [24] from France, were classified as high in quality. The inclusion of both phase I and II clinical trials (Naumann et al. [22] in a phase I/II trial and Rodrigues et al. [24] in a phase I trial) demonstrates the exploratory nature of these studies in evaluating safety, efficacy, and optimal dosing regimens for nivolumab. On the other hand, the studies by Tamura et al. [21] and Santin et al. [23], both phase II trials, further investigate the therapeutic potential of nivolumab with a focus on its clinical effectiveness and patient outcomes, providing valuable insights into its application in later stages of development.

All studies were rated high quality, two of them being developed in the United States (Naumann et al. [22] and Santin et al. [23]) and two other contributions from Japan (Tamura et al. [21]) and France (Rodrigues et al. [24]).

### 3.2. Patients’ Characteristics

The analysis of nivolumab in the four clinical trials described in Table 2 presents the characteristics of a total of 80 patients. Tamura et al. [21] involved 20 patients, with a median age of 50 years, ranging from 32 to 68. The comparison group in this study was between PD-L1 positive and PD-L1 negative patients, with a performance status showing 75% of patients at ECOG 0 and 25% at ECOG 1. Similarly, Naumann et al. [22] included 19 patients, with a slightly higher median age of 51 years, ranging from 28 to 75. This study differentiated between cervical cancer and vaginal-vulvar cancer patients, showing a diverse application of nivolumab across gynecological malignancies. The performance status distribution was 52.6% at ECOG 0 and 42.1% at ECOG 1, indicating a relatively good baseline functional status among the participants.

Santin et al. [23], with 25 patients, had the youngest median age group at 45 years, although the age range was not reported. The performance status here was slightly better balanced than in the previous studies, with 64% at ECOG 0 and 36% at ECOG 1, suggesting that a significant proportion of patients maintained a high level of activity despite advanced disease. Also, Rodrigues et al. [24] reported on 16 patients, providing an average age of 47.9 years, with a wide range spanning from 27 to 77 years. This study did not include a specific comparison group and had an evenly distributed performance status with 50% at ECOG 0 and 50% at ECOG 1, indicating a diverse patient functional status.

### 3.3. Disease Characteristics

In the trial by Tamura et al. [21], it was observed that the majority of patients (60%) were in the recurrent stage of the disease, indicating a challenging patient population with significant prior disease progression. Histology predominantly consisted of Squamous Cell Carcinoma (SCC) at 70%, with a smaller proportion of Adenocarcinoma (ACC) and Adenosquamous Carcinoma (ASC). The metastasis profile showed a diversity in disease spread, with 45% having fewer than two metastatic sites. Notably, 45% of patients were HPV 16–18 positive, and a significant 75% were PD-L1 positive, highlighting the potential for targeted immunotherapy. The high rates of prior radiotherapy (85%) and chemotherapy (100%) underscore the extent of the previous treatments these patients had undergone.

Naumann et al. [22] included patients predominantly in stages IVA-IVB (84.2%), with all patients having SCC, reflecting a highly uniform histological profile. Metastasis was commonly observed in lymph nodes (63.2%) and lungs (42.1%). The high HPV positivity rate (83.3%) and PD-L1 positivity (62.5%) suggest a significant immunotherapy target population. All patients received prior chemotherapy, and a large majority (89.5%) underwent radiotherapy, indicating a pre-treated population with advanced disease.

Santin et al. [23] presented a broader distribution of disease stages, with 60% in early stages I–II and 20% each in stages III and IV. The histological makeup was more varied than in Naumann et al. [22], with 60% SCC, 24% ACC, and 16% ASC. Metastasis was reported in 28% of patients, and a notable 77.3% were PD-L1 positive. The treatment history included high rates of radiotherapy (92%), some immunotherapy (8%), and surgery (68%), showing a diverse pre-treatment background among patients.

**Figure 2:**
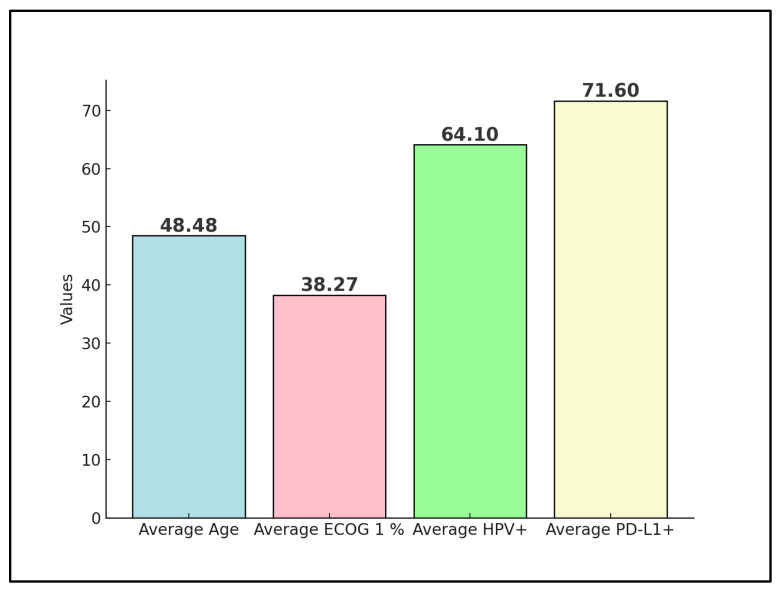
Aggregate values of disease characteristics among studies.

Rodrigues et al. [24] had a majority of patients in early stages (I–II: 62.5%), with SCC forming the bulk of histology (87.5%). No metastases were reported, indicating a potentially localized disease profile at the time of nivolumab treatment, as presented in Table 3. The HPV positivity rate was 64% (Figure 2), with all patients receiving concomitant chemotherapy, radiotherapy, and brachytherapy, suggesting a comprehensive treatment approach prior to or alongside nivolumab administration.

### 3.4. Outcomes

Tamura et al. [21] reported an Objective Response Rate (ORR) of 25% for PD-L1 positive patients and 0% for PD-L1 negative patients, with a Disease Control Rate (DCR) of 75%. The median follow-up time was 8.6 months, and the 6-month Progression-Free Survival (PFS) rates were 86% for PD-L1(+) versus 80% for PD-L1(–), with a median PFS of 5.5 months for PD-L1(+) versus 6.2 months. The study concluded that nivolumab demonstrated acceptable toxicity and clinical activity in advanced cervical cancer, highlighting the potential benefit of PD-L1 as a biomarker for response.

Naumann et al. [22] observed an ORR of 26.3% and a DCR of 68.4%, with a significant 15.8% of patients achieving a complete response. With a median follow-up time of 19.2 months, the median overall survival (OS) was reported at 21.9 months, and the 12-month PFS rate was 26.3%, with a median PFS of 5.1 months. These results underscore nivolumab’s safety and efficacy, showing promising survival outcomes in patients with advanced cervical cancer.

Santin et al. [23] utilized a different dosing regimen, leading to a median Stable Disease (SD) rate of 36% and a high rate of adverse events at 84%. The median OS was 14.5 months, with a median PFS at 3.5 months and 6-month PFS and OS rates at 16% and 78.4%, respectively. Despite the high incidence of adverse events, the study acknowledged nivolumab’s good safety profile but noted limited antitumor activity when used as monotherapy.

**Figure 3:**
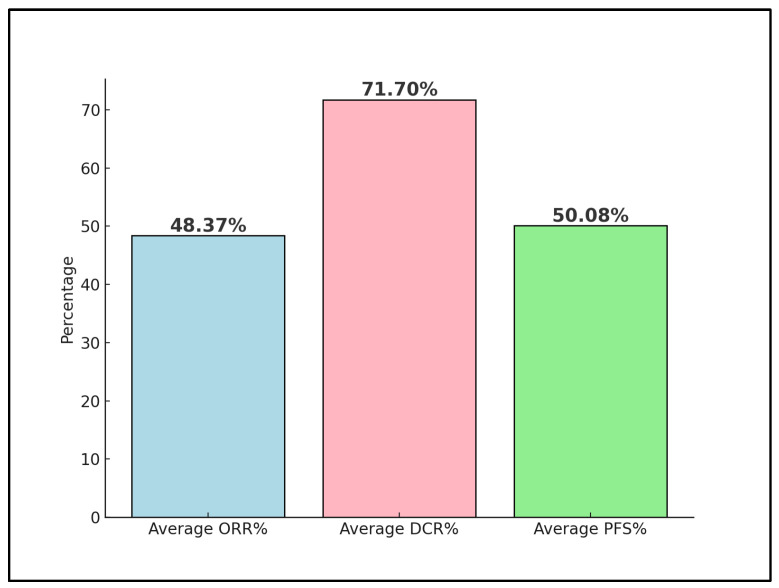
Nivolumab trials’ outcomes; ORR—Objective Response Rate; DCR—Disease Control Rate; PFS—Progression Free Survival.

Rodrigues et al. [24] reported an exceptional ORR of 93.8% and a median follow-up time of 23.8 months. Dose-limiting adverse events were reported in 20% of the patients. The 2-year PFS rate was 75%, with a median PFS not yet reached, indicating a potentially longer duration of benefit (Table 4). The study highlighted that nivolumab, when used in conjunction with concomitant chemoradiotherapy, is safe and exhibits promising PFS, suggesting a synergistic effect that enhances treatment outcomes (Figure 3).

Across these studies, the efficacy of nivolumab in treating advanced cervical cancer is evident, with the variations in the response rates and survival outcomes likely reflecting the differences in patient populations, treatment regimens, and PD-L1 status. The reported adverse events and the safety profile of nivolumab were generally favorable, except for a notable percentage of adverse events in the study by Santin et al. [23]. Rodrigues et al. [24] showed the most promising outcomes, particularly in ORR and PFS, indicating the potential for nivolumab as part of a combined treatment strategy. These findings collectively support the continued investigation and integration of nivolumab into treatment protocols for advanced cervical cancer, highlighting the importance of PD-L1 status and the potential benefits of combination therapies.

## 4. Discussion

### 4.1. Summary of Evidence

The clinical trials reviewed in our systematic analysis present compelling evidence of nivolumab’s role in managing advanced and metastatic cervical cancer. Across the four studies, nivolumab exhibited varying degrees of effectiveness, highlighted by the differential response rates and progression-free survival outcomes. The pooled ORR estimate of 48.37% indicates that when combining the data from all studies, on average, about 48.37% of patients treated with nivolumab in the context of advanced cervical cancer demonstrated a measurable response to the treatment. The studies by Tamura et al. [21] and Naumann et al. [22] showcased nivolumab’s potential, with objective response rates indicating a tangible benefit for a subset of patients. Particularly, the distinction in response based on PD-L1 status as reported by Tamura et al. [21] underscores the importance of biomarker-driven treatment strategies in optimizing patient outcomes. This biomarker’s predictive value is further supported by the favorable PFS rates observed, suggesting that PD-L1 positivity may enhance nivolumab’s therapeutic efficacy.

The integration of nivolumab into treatment regimens was further explored in the studies, revealing a good safety profile across diverse patient populations. Notably, Naumann et al. [22] demonstrated nivolumab’s efficacy with a median overall survival of 21.9 months and a 12-month PFS of 26.3%. These results are promising, considering the advanced disease stage of the patient cohort. However, Santin et al. [23] highlighted the challenges associated with nivolumab monotherapy, where a lower antitumor activity was observed alongside a significant rate of adverse events. This outcome suggests that while nivolumab holds therapeutic potential, its role may be more complex and context-dependent, necessitating further investigation to optimize its application.

Rodrigues et al. [24] provided an optimistic view of nivolumab’s application, achieving an ORR of 93.8% and an impressive 2-year PFS of 75%. This study’s outcomes, particularly the not-yet-reached median PFS, illustrate the immense promise of combining nivolumab with concomitant chemotherapy and radiotherapy. The synergistic effect observed suggests a pivotal role for nivolumab in enhancing the efficacy of existing treatment modalities, potentially redefining standard care practices for advanced cervical cancer.

The study conducted by Shieh et al. [25], despite identifying ten patients for the study, was notably excluded from the current systematic review due to the treatment of only one patient with nivolumab, while the other 9 were treated with pembrolizumab. Nevertheless, it is worth mentioning that a response rate of 70% was observed, alongside a median response duration of 21.0 months following a follow-up period of 20.7 months. Notably, patients exhibiting a PD-L1 combined positive score (CPS) ≥ 10 or a tumor mutation burden (TMB) ≥ 10 mut/Mb demonstrated higher response rates of 80% and 75%, respectively. The study reported a mean progression-free survival (PFS) of 20.2 months across the cohort, with several patients receiving treatment for more than 12 months. Interestingly, the response to immunotherapy was comparably positive in both platinum-sensitive and platinum-resistant patients, indicating no significant difference influenced by prior platinum sensitivity.

The case report by Baettig et al. [26], detailing the use of nivolumab in a patient with chemotherapy-resistant cervical cancer and documenting an isolated immune-related adverse event of vulvitis, provides an intriguing insight into the potential and challenges of immunotherapy in this disease context. This singular report highlights the remarkable efficacy of nivolumab as a third-line treatment, achieving persistent complete remission in a scenario where options were previously considered limited. The identification and successful management of vulvitis as a novel immune-related adverse event underlines the importance of vigilance and appropriate care in the administration of ICIs. Despite its exclusion from the systematic review due to its case-specific methodology, this report serves as a catalyst for future studies aimed at corroborating these findings within larger patient populations and refining treatment strategies for improved outcomes.

The proceedings presented by Devabhaktuni et al. [27] and Naumann et al. [28] were also excluded from this systematic review, although they offer valuable insights into the use of nivolumab in the treatment of recurrent/metastatic cervical cancer, each within distinct contexts and methodologies. Devabhaktuni’s retrospective study from a single tertiary center in India focused on low-dose nivolumab administration, demonstrating its effectiveness and manageable toxicity in a setting constrained by financial limitations, with 75% of patients having an ECOG performance status of ≤1. This approach significantly improved accessibility for patients in low- and middle-income countries, presenting a pragmatic option for reducing financial toxicity. Conversely, Naumann’s report on the CheckMate 358 trial explored the combination of nivolumab plus ipilimumab in two different dosing regimens, showing a clinical benefit across patients with advanced cervical cancer regardless of PD-L1 status or prior systemic therapies. The combination regimen, particularly Combo B, highlighted notable efficacy, especially in patients with previous systemic therapy, achieving a median overall survival of 25.4 months in patients with prior systemic therapy under Combo B. Despite the higher incidence of all/grade 3–4 treatment-related adverse events in both Combo A and B compared to the manageable toxicity in the low-dose nivolumab study, these findings collectively underscore the potential of nivolumab-based treatments in improving outcomes for patients with advanced cervical cancer, offering hope through both innovative dosing strategies and combination therapies.

Two other important study results were not included in this systematic review due to the nature of the publication deeming exclusion. Both abstracts by Nakamura et al. [29] and Oaknin et al. [30] explore the innovative use of nivolumab in cervical cancer, yet in different clinical settings and with varying methodologies, providing insightful data into the potential of immunotherapy. Nakamura et al.’s study focuses on locally advanced cervical cancer patients treated with concurrent chemoradiation therapy and nivolumab, reporting impressive overall response rates of 100% in cohort A and 93.3% in cohort B, with a 12-month progression-free survival rate of 100% among 29 evaluable patients for both cohorts. This study emphasizes nivolumab’s safety and efficacy in a pre- and co-administration setting with chemoradiation therapy. On the other hand, Oaknin et al. [30] delve into the realm of recurrent/metastatic cervical cancer, examining nivolumab alone and in combination with ipilimumab across different cohorts. They observed ORRs of 26.3% for nivolumab alone, and up to 38.4% for the pooled N1I3 cohort (nivolumab 1 mg/kg + ipilimumab 3 mg/kg), with a median OS ranging from 15.2 to 21.6 months across treatment arms, indicating significant efficacy and durability of response, albeit with manageable toxicity profiles, including a notable incidence of grade 3/4 immune-mediated adverse events.

The trial by Massarelli et al. [31] explored an innovative approach by combining the anti-PD-1 immune checkpoint antibody, nivolumab, with ISA 101, a synthetic long-peptide HPV-16 vaccine, aiming to enhance the treatment efficacy in patients with incurable HPV-16–positive cancer. Despite only one patient with cervical cancer being included, making its direct applicability to cervical cancer management limited within this study, the findings provide a glimpse into potential multi-modal immunotherapeutic strategies. The overall response rate of 33% among the 24 patients, significantly higher than the 16% to 22% typically observed with PD-1 inhibitors alone, alongside a median overall survival of 17.5 months, underscores the promising synergy between vaccination and checkpoint inhibition. However, the median progression-free survival of only 2.7 months indicates the complex dynamics between the initial response and durable control of the disease. Given the manageable grades 3 to 4 toxicity in two patients, this combination therapy appears feasible, warranting further exploration in a randomized clinical trial to validate these preliminary findings and to determine the precise contribution of the HPV-16 vaccine to the tumoricidal effects of PD-1 inhibition, potentially revolutionizing the treatment paradigm for HPV-driven cancers, including cervical cancer.

The trial by Pouyiourou et al. [32] investigated the efficacy of nivolumab and ipilimumab in patients with cancer of unknown primary, focusing on the distinction between high tumor mutational burden (TMB) and low TMB as a predictive marker for response to immunotherapy. This Phase II trial, although terminated prematurely, provided critical insights into the role of TMB in guiding immunotherapy decisions for CUP, a condition notoriously difficult to treat due to its elusive origin. Among the 31 evaluable patients, patients with high TMB showed a remarkable response rate of 60% and a notably longer PFS and OS at 18.3 months, compared to just 2.4 and 3.6 months, respectively, in the low TMB group, despite the overall response rate standing at 16%. Furthermore, the trial highlighted the utility of assessing circulating tumor DNA dynamics as an innovative approach to identifying patients likely to benefit from treatment, even beyond initial radiologic assessments. However, the high incidence of severe immune-related adverse events in 29% of cases indicates a need for the careful management and monitoring of patients undergoing this treatment combination, balancing the potential for significant therapeutic benefits against the risks of adverse effects. Nevertheless, this study was excluded from the current systematic review due to the multiple sites of the primary cancer in the studied patients.

In the clinical trial by Wolf et al. [33], involving a cohort of metastatic cervical cancer patients treated with ICIs as second-line therapy, six patients were followed for up to 40 months, revealing significant long-term benefits. However, it was not included in the systematic analysis because it is unclear which patients received nivolumab or pembrolizumab due to the blinding protocol. Moreover, the trial is still ongoing and only some preliminary data were presented. Six patients achieved a complete response, with three improving from an initial partial response (with a mean of 3 months) to complete response after a mean time of 16 months after prolonged treatment. Four patients discontinued the treatment; among these, two who experienced asymptomatic recurrences were successfully re-initiated on ICIs, achieving complete and partial response upon retreatment. This study suggests the durability of ICI efficacy, highlighting that responses can deepen over time with prolonged treatment and can be effectively regained with retreatment. Moreover, the variation in the combined positive score among patients, particularly higher levels in those achieving prolonged remission off treatment, provides a critical insight into potential biomarkers for predicting treatment outcomes.

One aspect that might influence the outcomes is the co-infection with high-risk HPV strains. Although it could be hypothesized that co-infection is a worsening factor, the study by Wu et al. [34] found in their Chinese cohort that HPV16/18-positive women co-infected with other high-risk HPV types had a lower risk of developing high-grade cervical intraepithelial neoplasia (CIN3+), with an odds ratio of 0.637 (95% CI = 0.493–0.822), indicating a significantly decreased risk compared to those with a single HPV16 infection. In contrast, Senapati et al.’s study [35] within the Indian population highlighted the elevated risk associated with multiple HPV genotypes, particularly those not covered by the quadrivalent vaccine, showing a 2.94-fold increased risk of cervical carcinoma (OR = 2.94, 95% CI = 1.48–5.80).

When assessing a new potential treatment, other variables such as the number needed to treat (NNT) and number needed to harm (NNH) are also important. In one study [36] on nivolumab’s integration with chemotherapy for treating advanced/metastatic gastric, gastroesophageal junction cancer, and esophageal adenocarcinoma, the calculated number needed to treat (NNT) for improving the overall survival (OS), progression-free survival, and objective response rate (ORR) indicates significant patient benefits, especially noted in a subgroup with PD-L1 CPS ≥ 5, suggesting enhanced effectiveness in patients with higher PD-L1 expression. With NNTs for OS at 15.15 and 12.05 over 1 and 2 years, respectively, and a notably lower NNT for ORR at 8.95, the advantages of adding nivolumab to standard chemotherapy are clear. Conversely, the number needed to harm (NNH) for experiencing grade ≥ 3 treatment-related adverse events (TEAEs) stands at 7.02 over one year, indicating a relatively favorable safety profile.

Overall, the existing literature underlines the critical role of PD-L1 in predicting the efficacy of immunotherapies in different cancer types, suggesting the necessity for tailored treatment approaches based on PD-L1 status. As such, the study by Schmidt et al. [37] revealed that among cervical cancer treatments, pembrolizumab combined with chemotherapy and bevacizumab significantly increases overall survival, highlighting the importance of selecting patients based on PD-L1 status due to the lack of response in PD-L1 negative individuals. Meanwhile, Arak et al. [38] provided crucial insights into non-small cell lung cancer, showing that high PD-L1 expression—found in 37% of patients—correlates with more aggressive disease and reduced overall survival (24 months in PD-L1 positive patients versus 48 months in negatives), emphasizing the prognostic significance of PD-L1.

Critically analyzing these results, it is evident that nivolumab’s effectiveness in advanced cervical cancer is multifaceted, influenced by factors such as PD-L1 status, the disease stage, and the combination with other treatments. While the safety profile is generally favorable, the variability in antitumor activity and the occurrence of adverse events highlight the need for precise patient selection and tailored treatment approaches. Future research should focus on elucidating the mechanisms underlying the variable response rates, exploring biomarkers that can predict treatment success, and defining optimal combination therapies that can maximize patient benefits. The promising outcomes observed, particularly in studies like Rodrigues et al. [24], underscore nivolumab’s potential as a transformative agent in the treatment landscape of advanced and metastatic cervical cancer, warranting further exploration and validation in larger, randomized clinical trials.

### 4.2. Limitations

This systematic review, despite its comprehensive methodology and adherence to PRISMA guidelines, faces several specific limitations impacting its conclusions. One major constraint is the exclusion of studies presented solely as abstracts or proceedings, which could hold significant preliminary data on nivolumab’s effectiveness in advanced cervical cancer. This exclusion criterion might have led to the omission of recent findings and ongoing trials that have yet to culminate in full-text publications, thereby potentially limiting the breadth of analyzed data. The study’s reliance on peer-reviewed articles also means that gray literature, including important clinical trial updates and conference presentations that could provide additional insights into nivolumab’s use, was not considered, introducing a publication bias. Furthermore, the heterogeneity in the study designs, patient populations, and outcome measures among the included studies complicates the aggregation and comparison of data, potentially affecting the synthesis of evidence. This variability suggests that differences in study methodologies and patient demographics may influence the observed effects of nivolumab, hindering the synthesis of evidence and possibly affecting the generalizability and interpretation of the results. Lastly, another limitation stands as a barrier to conducting a meta-analysis due to the heterogeneity of the study designs, variability in patient populations and treatment regimens, and the diversity of the outcome measures reported across clinical trials.

## 5. Conclusions

The systematic review highlights nivolumab’s promising role in the treatment of advanced and metastatic cervical cancer, underscoring its potential to improve outcomes in a notably difficult-to-treat patient population. The variability in response rates and the significant influence of PD-L1 positivity on therapeutic effectiveness emphasize the need for personalized treatment strategies, particularly considering the potential benefits of combination therapies. These findings underline the importance of further research to optimize treatment protocols and identify predictive biomarkers, ultimately aiming to enhance the clinical utility of nivolumab and improve patient survival and quality of life in the context of advanced cervical cancer.

## Figures and Tables

**Table 1:** Study characteristics.

Study and Author	Country	Study Year	Study Design	Study Quality
1 [21] Tamura et al.	Japan	2019	RCT (phase II)	High
2 [22] Naumann et al.	United States	2019	RCT (phase I/II)	High
3 [23] Santin et al.	United States	2019	RCT (phase II)	High
4 [24] Rodrigues et al.	France	2023	RCT (phase I)	High

RCT—Randomized Clinical Trial.

**Table 2:** Patients’ characteristics.

Study Number	Sample Size	Age (Years)	Comparison Group	Performance Status
1 [21] Tamura et al.	20	Median: 50 Range: 32–68	PD-L1 positive vs. PD-L1 negative	ECOG 0: 75% ECOG 1: 25%
2 [22] Naumann et al.	19	Median: 51 Range: 28–75	Cervical cancer vs. Vaginal-vulvar cancer	ECOG 0: 52.6% ECOG 1: 42.1%
3 [23] Santin et al.	25	Median: 45	NR	ECOG 0: 64% ECOG 1: 36%
4 [24] Rodrigues et al.	16	Mean: 47.9 Range: 27–77	NR	ECOG 0: 50% ECOG 1: 50%

NR—Not Reported; ECOG—Eastern Cooperative Oncology Group; PD-L—Programmed Death-Ligand.

**Table 3:** Disease characteristics.

Study Number	Stage	Histology	Metastases	HPV/PD-L Status	Prior Treatment
1 [21] Tamura et al.	III: 5% IV: 35% Recurrent: 60%	SCC: 70% ACC: 25% ASC: 5%	<2: 45% 2: 35% >2: 20%	HPV 16–18 positive: 45% PD-L1 positive: 75%	Radiotherapy: 85% Chemotherapy: 100%
2 [22] Naumann et al.	IIB: 5.3% IIIB–IIIC: 10.5% IVA–IVB: 84.2%	SCC: 100%	Lymph nodes: 63.2% Lungs: 42.1% Pelvis: 26.3% Uterus: 15.8% Peritoneum: 10.5% Bones: 10.5%	HPV 6,11,16,18,33 positive: 83.3% PD-L1 positive: 62.5%	Radiotherapy: 89.5% Chemotherapy: 100%
3 [23] Santin et al.	I–II: 60% III: 20% IV: 20%	SCC: 60% ACC: 24% ASC: 16%	28% of patients	PD-L1 positive: 77.3%	Radiotherapy: 92% Immunotherapy: 8% Surgery: 68%
4 [24] Rodrigues et al.	I–II: 62.5% III: 31.3% IV: 6.2%	SCC: 87.5% ACC: 12.5%	0%	HPV 16–18 positive: 64%	Concomitant chemotherapy: 100% Radiotherapy: 100% Brachytherapy: 100%

SCC—Squamous Cell Carcinoma; ACC—Adenocarcinoma; ASC—Adenosquamous Carcinoma; HPV—Human Papilloma Virus; PD-L—Programmed Death-Ligand.

**Table 4:** Summary of outcomes.

Risk Factors	Treatment/Dose	Follow-Up	Survival	Conclusions
1 [21] Tamura et al.	240 mg at 2-week intervals Median duration of treatment: 5.4 months	ORR: 25% PD-L1(+) vs. 0% PD-L1(−) DCR: 75% Median follow-up time: 8.6 months	6-month PFS: 86% PD-L1(+) vs. 80% PD-L1(−) Median PFS: 5.5 months PD-L1(+) vs. 6.2 months	Nivolumab showed acceptable toxicity in all cohorts, with evidence of clinical activity in advanced cervical cancer.
2 [22] Naumann et al.	240 mg at 2-week intervals Median duration of treatment: 5.6 months	ORR: 26.3% DCR: 68.4% Complete response: 15.8% Median follow-up time: 19.2 months	Median OS: 21.9 months 12-month PFS: 26.3% Median PFS: 5.1 months	Nivolumab proved a good safety record and efficacy in advanced cervical cancer.
3 [23] Santin et al.	Four doses of IV nivolumab (3 mg/kg every 2 weeks), followed by an additional 42 doses of 3 mg/kg every 2 weeks for a maximum of 46 doses	Median ORR: 5.7 months ORR: 36% Adverse events: 84%	Median OS: 14.5 months Median PFS: 3.5 months 6-month PFS: 16% 6-month OS: 78.4%	Nivolumab proved a good safety record besides the number of adverse events. Low antitumor activity as monotherapy.
4 [24] Rodrigues et al.	240 mg at 2-week intervals	ORR: 93.8% Median follow-up time: 23.8 months Dose-limiting adverse events: 20%	2-years PFS: 75% Median PFS: has not been reached	Nivolumab associated with concomitant chemoradiotherapy is safe and shows promising PFS.

PFS—Progression-Free Survival; OS—Overall Survival; ORR—Objective Response Rate; PD-L—Programmed Death-Ligand; DCR – Disease Control Rate.
